# Early phase studies in India: Are we too early to explore?

**DOI:** 10.4103/0253-7613.44149

**Published:** 2008-10

**Authors:** Anupama Ramkumar

**Affiliations:** Arkus Clinical Trial Support Solutions, 17, Pushpak, Behind Swagat Plaza-2, Ambli, Ahmedabad-380 058, India. E-mail: anupama.ramkumar@gmail.com

Early phase studies are exploratory, first-in-man studies that are conducted most often (but not always) in healthy volunteers and are critical to the development of a potential new drug. The objective of these studies is to establish proof of safety of the new molecule, as well as its pharmacokinetics in healthy volunteers and often other special study populations, before proceeding on to clinical trials with patients. These studies are an essential step before taking a ‘go/no-go’ decision for further development.

The current economic environment forces the pharmaceutical industry to do more at a faster rate and at less cost, and bring new compounds early and efficiently to the market. Several midsize to small pharma companies abroad, view India as a potential area for the conduct of these studies. However, current Indian regulations do not allow first-in-man dosing studies of foreign molecules to be conducted in Indian populations. Phase 1 trials (as they are also known) can however be conducted for drugs developed in India, of which there are not many. For the last few years there has been intense discussion between the clinical research industry and the Indian regulatory agencies regarding the opening of the doors for the conduct of phase 1 studies in India for foreign molecules. Recent reports suggest that the Indian regulatory authority is close to taking a decision in this regard and may soon allow the conduct of these trials by contract research organizations in India.[1]

In the wake of the international media attention that TeGenero received in March 2006 after administering TGN1412 to six healthy volunteers who suffered a nearly fatal ‘cytokine storm,’ several countries intensely scrutinized how first-in-human phase I trials are conducted and introduced several scientific, ethical, and regulatory measures to make these trials safer.[2] Closer home, the revelation that 49 infants died during clinical trials (late phase trials) sparked a lot of social debate and anger among the nation's congress, with some calling for all studies to be stopped while investigations take place; this is despite the fact that the infant mortality rate (IMR) among the participants in the trial was below the national average. A large section of society believes that ‘the unquestionable ease with which clinical trials can be conducted in India makes international agencies first test their products on the Indian population.’ This viewpoint appeared to have won support in some parts of the Indian congress, with a congressman stating: ‘The practice of using infants like guinea-pigs for drug testing must end.’[3] With sentiments such as these and the possibility of the occurrence of incidents such as TeGenero, coupled to the fact that the safety of the human volunteer is the research community's combined social, moral, and scientific responsibility, it is time to ask ourselves: ‘Are we ready for these studies yet?’

Issues regarding regulatory preparedness, availability of niche scientific expertise as well as experience, infrastructure and finally investment in education, both of the stakeholders as well as society take paramount importance. Early phase study protocols are designed based on the information generated from animal and other preclinical studies, and the ability to understand, review, and apply this data to the human studies becomes imperative. Expertise and experience in this area is limited in the country at present, both amongst the regulatory as well as the contract research organisation (CRD) community. With the reluctance to invest in the training of personnel, whether in India or at facilities abroad, this remains a need waiting to be fulfilled. There have been recent positive moves in the regulatory agencies in India, including central drugs standard control organisation (CDSCO), to train and educate their staff to inspect CROs; however, prior to that, the ability to grant approval for the studies will depend upon the availability of in-house expertise for evaluating the study design and the safety aspects of studies, as well as the capability of the CRO to conduct them. The recent proposal towards inspection and registration of CROs will bear fruit only when the regulatory inspectors are trained to evaluate these sites for study conduct. And all this in keeping with an expectation of quick turnaround times for regulatory approval and minimum regulatory hurdles.[4]

The infrastructure requirements (apart from having a housing facility where healthy volunteer studies can be conducted) include a fully equipped, multi-bedded emergency handling unit whose staff understand the special requirements of these studies and are also well trained in advanced emergency resuscitative measures. Several bioequivalence centers with extensive experience with healthy volunteer studies have upgraded some of their infrastructure and facilities to meet the needs of early phase studies. This alone may not be sufficient as there is a great deal of difference in the risk assessment and risk management in bioequivalence *vs* first-in-man studies, and the only way to overcome this is education and training. Designating an appropriately qualified ‘Principal Investigator’ for early phase studies is still something that many CROs find difficult to implement because there is a lack of clear understanding and consensus on what constitutes ‘appropriately qualified’ and also because there is a dearth of such investigators. Trained teams are the next necessity and, in their training, special emphasis needs to be laid on emergency procedures, including a formal readiness assessment. These measures can be effected only when there is an informed awareness about the responsibilities and expectations that come with being part of a early phase team which, considering the current lack of cumulative experience secondary to the lack of opportunity, can only be realized through knowledge sharing forums and focused training. Given the high attrition rates in the industry, few companies are ready to invest considerable time and resources on training people till they can see business benefits in the near future, thus binding themselves in a typical ‘Catch 22’ situation.

Creation of awareness among the ethics committees, the media, and society in general is also an important step. Ethics committees are the immediate watchdogs for the rights and safety of the participants in the study and, importantly, also one of the bodies that need to approve the trial prior to its commencement. Specific training in the scientific as well ethical aspects of the early phase studies would go a long way in mitigating some of the concerns of the ethics committees as well as in arming them with better means of overseeing the progress of the trials; this would also be of help during subsequent decision making on escalating doses. Media and society, more often than not, perceive participants in research (sometimes justifiably) as ‘human guinea pigs.’ In the western world there are patient support groups and several not-for-profit organizations that act on behalf of the research participants. Members of these bodies are quite often invited to participate in the building of the research process, thus aiding in winning society's confidence in these studies. Emulating measures such as these would not only create a positive awareness but would also instill confidence in the public regarding the necessity and conduct of this phase of drug development. The 'Points to consider 'document and recommendations compiled by the ABPI/BIA Task force, which was set up in 2006 to provide industry input to the expert working group in U.K after the TeGenero episode can be used as a kind of blue print in this regard by the industry for phase 1 studies.[5] Essentially, until we can ensure that we have the capability for safety evaluation right from the stage of preclinical data collection to protocol design and review, until we can provide focused education and training of all the stake holders, and until we have a regulatory framework that is proactively involved as a watchdog, we are just around the corner, but have not yet arrived in our quest for the conduct of early phase studies.
